# Efficacy of immune checkpoint inhibitor therapy in EGFR mutation-positive patients with NSCLC and brain metastases who have failed EGFR-TKI therapy

**DOI:** 10.3389/fimmu.2022.955944

**Published:** 2022-09-27

**Authors:** Shujie Zhou, Fei Ren, Xiangjiao Meng

**Affiliations:** ^1^ Department of Radiation Oncology, Shandong Cancer Hospital and Institute, Cheeloo College of Medicine, Shandong University, Jinan, Shandong, China; ^2^ Department of Radiation Oncology, Shandong Cancer Hospital and Institute, Shandong First Medical University and Shandong Academy of Medical Sciences, Jinan, Shandong, China

**Keywords:** immune checkpoint inhibitors (ICIs), epidermal growth factor receptor (EGFR), non-small cell lung cancer (NSCLC), brain metastases (BMs), efficacy, prognosis

## Abstract

**Background:**

Few treatment options are available for brain metastases (BMs) in EGFR-mutant non-small cell lung cancer (NSCLC) that progress with prior EGFR tyrosine kinase inhibitor (EGFR-TKI) therapy. This study aimed to evaluate the efficacy of immune checkpoint inhibitor (ICI) therapy in these patients.

**Methods:**

NSCLC patients with confirmed sensitive EGFR mutations and BMs were retrospectively reviewed. All patients experienced failure of EGFR-TKI therapy and were divided into two cohorts based on subsequent treatment. Cohort 1 included patients who received ICI therapy, while cohort 2 included patients treated with chemotherapy. Overall and intracranial objective response rates (ORRs) were used to evaluate the treatment response. Overall and intacranial progression-free survival (PFS) were calculated by Kaplan−Meier analysis and compared with the log-rank test. Univariate and multivariate Cox analyses were used to identify prognostic factors.

**Results:**

A total of 53 patients treated with ICI therapy and 40 patients treated with chemotherapy were included in cohorts 1 and 2, respectively. In cohort 1, the overall ORR was 20.8%, with a median overall PFS of 4.2 months. The median intracranial PFS was 5.1 months. Of the 38 patients with measurable intracranial lesions, the intracranial ORR was 21.0%. Patients who received ICI combined with chemotherapy had the highest intracranial ORR of 37.5%. Compared to patients treated with chemotherapy in cohort 2, patients receiving ICI combined with chemotherapy had both longer intracranial PFS (6.4 *vs*. 5.1 months, p = 0.110) and overall PFS (6.2 *vs*. 4.6 months, p = 0.054), and these differences approached statistical significance. Univariate and multivariate Cox analyses demonstrated that high disease burden (p = 0.019), prior third-generation EGFR-TKI therapy (p = 0.019), and a poor lung immune prognostic index (LIPI) (p = 0.012) were independent negative predicators of overall PFS and that multiple BMs were negatively correlated with intracranial PFS among patients treated with ICI therapy.

**Conclusions:**

Our results suggested that ICI combined with chemotherapy had potent intracranial efficacy and may be a promising treatment candidate in EGFR-mutant NSCLC patients with BMs for whom prior EGFR-TKI therapy failed.

## Introduction

Non-small cell lung cancer (NSCLC) accounts for approximately 85% of all lung cancers with the highest number of cases of brain metastases (BMs) which lead to extremely poor prognosis (1). It is estimated that 20% to 40% of NSCLC patients with NSCLC will develop BMs after diagnosis (1–3). Patients with epidermal growth factor receptor (EGFR) mutations are associated with increased incidence of BMs compared with wild-type patients (27.4% *vs*. 14.5%, p = 0.009), suggesting that this genetic alteration is an important risk factor for developing BMs (4–6). EGFR-tyrosine kinase inhibitor (EGFR-TKI) therapy is the first-line treatment option in patients with EGFR mutations with metastases. After the failure of front-line TKIs, a small subset of patients can receive third-generation EGFR-TKI therapy if they have the T790M mutation and do not receive the third-generation drug before. The remaining patients without the T790M mutation are candidates only for chemotherapy, contributing to a relatively short median PFS of approximately 4–5 months (7, 8). Intracranial radiotherapy is the primary local treatment for patients with advanced NSCLC with EGFR mutations and BMs; however, most patients eventually experience disease progression in intracranial lesions. In addition, the necessity of implementing brain radiotherapy for patients with asymptomatic and stable BMs deserves special consideration, as brain radiotherapy may cause cognitive decline. Therefore, seeking new therapeutic strategies for those patients who experienced failure of EGFR-TKI therapy remains a challenge.

In recent years, immune checkpoint inhibitor (ICI) targeting programmed death-1 (PD-1) and PD-1 ligand (PD-L1) has revolutionized cancer treatment by harnessing the power of the immune system, and these ICIs dramatically improve the clinical outcomes of advanced NSCLC patients without driver mutations (9–12). The efficacy of ICI therapy was also observed in a subgroup of patients with BMs (13–16). A pooled analysis of three randomized studies showed that the objective response rate (ORR) and duration of response were 39.0% and 11.3 months, respectively, in NSCLC patients with BMs treated with ICI plus chemotherapy, while they were only 19.7% and 6.8 months in patients treated with chemotherapy alone (15). Moreover, one prospective study provided evidence supporting the use of PD-1 inhibitors in PD-L1-positive NSCLC patients with untreated BMs, with an overall survival (OS) of 9.9 months (16). These results justify the rational use of ICI therapy in the treatment of patients with BMs.

However, several studies suggest that EGFR mutations are associated with a poor response to ICI monotherapy in NSCLC (17–20). Patients with EGFR mutations who received PD-1 inhibitors had a low ORR of 14% and short median progression-free survival (PFS) of 1.8 months, while these values were 30% and 8.8 months in patients with wild-type EGFR (20). Nevertheless, this situation can be improved when ICIs are combined with other therapeutic modalities. Recently, a phase II study reported that patients with EGFR mutations receiving a PD-1 inhibitor plus chemotherapy had an ORR of 50% and a median PFS of 7.0 months after resistance to EGFR-TKI therapy (21). Moreover, the updated data of IMPOWER 150 have shown that the ORR was 73.5% and the median PFS was 10.2 months in patients treated with ICI triple therapy of a PD-L1 inhibitor plus chemotherapy plus antiangiogenic therapy (22). Although ICI therapy has promising efficacy in the treatment of EGFR-mutant NSCLC that progressed with prior EGFR-TKI therapy, little is known about the central nervous system (CNS) activities in the subpopulation with BMs.

In this retrospective study, we evaluated the efficacy of ICI therapy in EGFR-mutant NSCLC patients with BMs who failed prior EGFR-TKI therapy. Additionally, patients treated with salvage chemotherapy were enrolled to compare survival with that of patients treated with ICI therapy to determine whether ICI therapy is a promising treatment candidate.

## Materials and methods

This retrospective study was approved by the Ethics Review Board of Shandong Cancer Hospital and Institute and conforms to the provisions of the Declaration of Helsinki. This study was a retrospective analysis and did not require informed consent from patients.

### Patients

**Figure 1:**
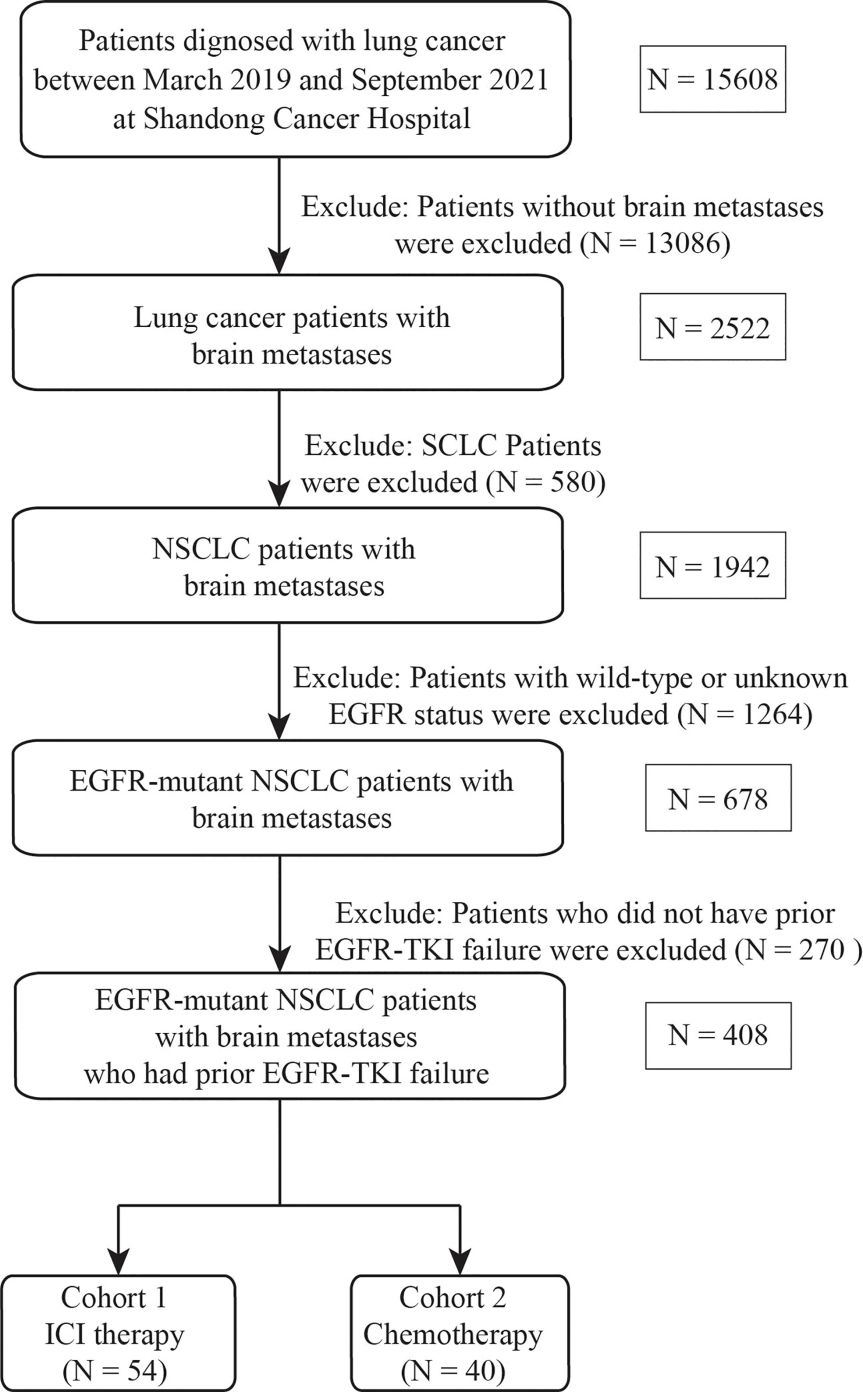
Flowchart of the screening procedure. EGFR, epidermal growth factor receptor; SCLC, small cell lung cancer, NSCLC, non-small cell lung cancer, TKI, tyrosine kinase inhibitor; ICI, immune checkpoint inhibitor.

All patients hospitalized in Shandong Cancer Hospital and Institute between March 2019 and September 2021 were retrospectively reviewed. EGFR-mutant NSCLC patients with BMs who received ICI, including pembrolizumab, nivolumab, sintilimab, camrelizumab, tislelizumab, and atezolizumab, were enrolled. The inclusion criteria were as follows: 1) pathological or cytological diagnosis of NSCLC; 2) BMs diagnosed by contrast brain magnetic resonance imaging (MRI) or computed tomography (CT) scan; 3) NSCLC with EGFR-sensitive mutations; and 4) experiencing failure of EGFR-TKI therapy. Patients were excluded if they received ICI therapy prior to the diagnosis of BMs and lacked baseline and at least one follow-up imaging scan. In addition, a cohort of patients treated with salvage chemotherapy were included using the same criteria to serve as comparative controls. The study flowchart is depicted in **Figure 1**.

In the study, we retrospectively collected the tumor and patient characteristics obtained from electronic medical records. We evaluated the efficacy of ICI therapy based on the combination regimens, which can be divided into ICI monotherapy, ICI plus chemotherapy, ICI plus antiangiogenic therapy, and ICI plus chemotherapy plus antiangiogenic therapy. We also conducted a comparative analysis between the groups receiving ICI plus chemotherapy *vs*. chemotherapy alone. Moreover, we performed univariate and multivariate Cox regression analyses to determine predictors of PFS. The data cutoff date was 31 March 2022.

### Study endpoints

The data were collected and analyzed according to the Response Evaluation Criteria in Solid Tumors version 1.1 (RECIST v1.1). Treatment response was divided into complete response (CR), partial response (PR), stable disease (SD), and progressive disease (PD). The overall response rate (ORR) was defined as the proportion of patients who had a CR/PR of any metastasis (considering both brain and extracerebral lesions), and the disease control rate (DCR) was defined as the proportion of patients with a CR/PR/SD. The intracranial ORR and DCR were calculated based on brain lesions. Intracranial progression-free survival (PFS) was defined as the date of the start of ICI therapy to the date of progression of intracranial lesions, death, or censoring on the date of the last imaging. Patients who had extracranial progression first were not included in the analysis of intracranial PFS. Overall PFS was defined from the start of ICI therapy to the occurrence of intracranial or extracranial progression or death or was censored on the date of the last imaging.

### Statistical analysis

Descriptive summaries were created for demographic and clinical variables. PFS was calculated by Kaplan–Meier survival analysis. Log-rank tests were used to compare the survival between groups. Univariate Cox regression and multivariate Cox regression were used to examine the association between clinical factors and PFS. A value of p < 0.05 was considered statistically significant. Variables with a value of p < 0.15 in univariate analyses were selected for multivariate analysis. All analyses were performed using R version 4.05.

## Results

### Patient characteristics

We finally identified 53 EGFR mutation-positive NSCLC patients with BMs who were treated with ICI therapy. The median follow-up was 6.9 months. The baseline clinical and pathological features are summarized in **Table 1**. The majority of patients were younger than 60 years (67.9%) and had an Eastern Cooperative Oncology Group (ECOG) performance status of 0–1 (88.7%), no smoking history (86.8%), a histological diagnosis of adenocarcinoma (90.7%), negative/unknown PD-L1 expression (81.1%), and three or more metastatic organs (53.2%). Forty-six (86.8) patients received multiline prior systemic therapies, whereas 23 (43.4%) received prior intracranial RT. For brain lesions, 11 patients (21.2%) had symptomatic BMs and 28 (52.8%) had multiple BMs.

**Table 1:** Clinical characteristics of the study population.

Characteristic	N (%)
**Age at diagnosis** <60 ≥60	36 (67.9) 17 (32.1)
**Sex** Male Female	27 (50.9) 26 (49.1)
**ECOG-PS** 0-1 2	47 (88.7) 6 (11.3)
**Smoking history** Never Smoked	46 (86.8) 7 (13.2)
**Histology** Adenocarcinoma Non-adenocarcinoma	50 (94.3) 3 (5.7)
**Molecular genotype** EGFR 19del EGFR 21L858R EGFR rare mutation	22 (41.5) 23 (43.4) 8 (15.1)
**Acquired T790M mutation** No or unknown Yes	40 (75.5) 13 (24.5)
**PD-L1 expression** Negative or unknown 1%-49% ≥50%	43 (81.1) 5 (9.4) 5 (9.4)
**High disease burden** No Yes	22 (46.8) 25 (53.2)
**Number of BMs** Single Multiple	25 (47.2) 28 (52.8)
**Symptomatic BMs** No Yes	41 (78.8) 11 (21.2)
**Prior lines of systemic therapy** 1 ≥2	7 (13.2) 46 (86.8)
**Prior intracranial RT** No Yes	30 (56.6) 23 (43.4)
**Prior TKI response time** <10 months ≥10 months	26 (49.1) 27 (50.9)
**Prior third-generation TKI treatment** No Yes	24 (45.3) 29 (54.7)
**Corticosteroid use at start of ICI treatment** No Yes	24 (45.3) 29 (54.7)
**Combination modalities** ICI monotherapy ICI plus chemotherapy ICI plus anti-angiogenesis ICI plus chemotherapy plus anti-angiogenesis	4 (7.5) 19 (35.8) 12 (22.6) 18 (34.0)
**Concurrent intracranial RT** No Yes	43 (81.1) 10 (18.9)
**Concurrent extracranial RT** No Yes	43 (81.1) 10 (18.9)
**dNLR** <3 ≥3	41 (77.4) 12 (22.6)
**LDH** ** <**ULN ≥ULN	31 (58.5) 22 (41.5)
**LIPI** Good (0) Intermediate (1) Poor (2)	28 (52.8) 16 (30.2) 9 (17.0)

EGFR, epidermal growth factor receptor; ECOG-PS, Eastern Cooperative Oncology Group performance status; PD-L1, programmed death-1 ligand; BMs, brain metastases; RT, radiotherapy; TKI, tyrosine kinase inhibitor; ICI, immune checkpoint inhibitor; dNLR, derived neutrophil-to-lymphocyte ratio; LDH, lactate dehydrogenase; ULN, upper limit of normal; LIPI, immune prognostic index.

Twenty-two (41.5%) patients had EGFR 19del mutations, 23 (43.4%) had EGFR 21L858R mutations, and eight (15.1%) had rare EGFR mutations, including three EGFR 18G719X, three EGFR 20S768I, and two EGFR 21L861Q mutations. All patients experienced failure of EGFR-TKI therapy; among them, 29 (54.7%) had previously used third-generation EGFR-TKI therapy, and 26 (49.1%) had TKI response durations of less than 10 months. Regarding the treatment modalities, 19 (35.8%) received ICI plus chemotherapy, 12 (22.6%) were treated together with antiangiogenic therapy, 18 (34.0%) received immunotherapy plus chemotherapy plus antiangiogenic therapy, and only 4 (7.5%) received monotherapy. In addition, 10 (18.9%) received concurrent intracranial RT with ICI therapy. The lung immune prognostic index (LIPI) was calculated based on the baseline derived neutrophil-to-lymphocyte ratio (dNLR) and lactate dehydrogenase (LDH), as previously reported (23). Overall, 28 (52.8%) had an LIPI = 0 (good), 16 (30.2%) had an LIPI = 1 (intermediate), and 9 (17.0%) had an LIPI = 2 (poor).

### Assessment of efficacy

**Figure 2:**
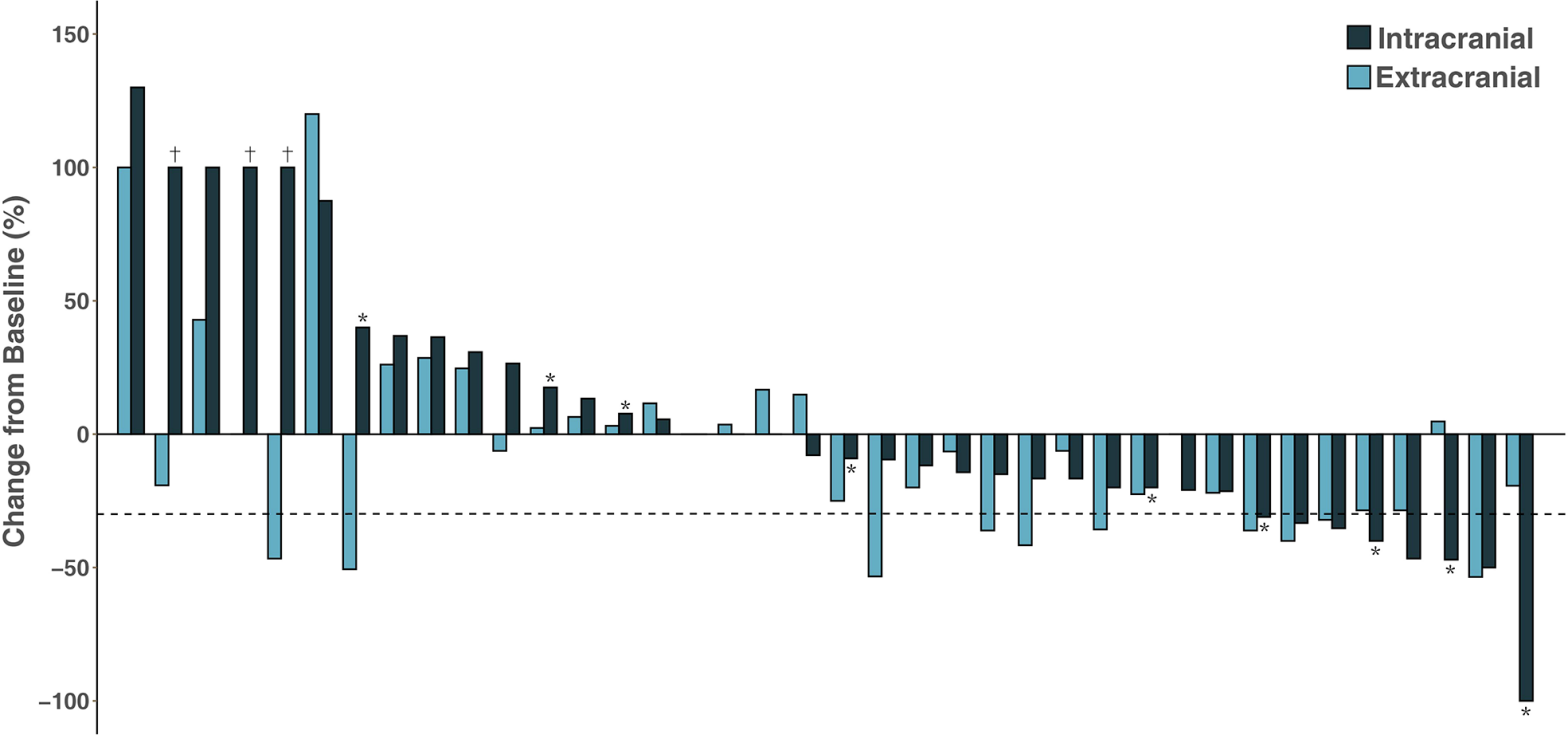
Waterfall plot of intracranial and extracranial change patients with measurable intracranial lesions. ^†^Disease progression due to the development of new lesions; *Patients receiving concurrent intracranial radiotherapy.

Among the 53 patients, none had a CR, 11 had a PR (20.8%), 27 (50.9%) had SD, and 15 (28.3%) experienced PD; thus, the ORR was 20.8% and the DCR was 71.7%. The patients who underwent the combination of ICI and chemotherapy had the highest ORR of 36.8%, while no response was observed in patients receiving ICI monotherapy and ICI plus antiangiogenic therapy. Of the 38 patients with measurable intracranial lesions, the intracranial ORR was (21.0%) (one CR and seven PRs). Patients treated with ICI plus chemotherapy had the highest CNS response rate of 37.5% compared to other three treatment strategies. Nine patients with measurable BMs received concurrent intracranial RT with ICI therapy; the intracranial ORR was 44.4% (one CR and three PRs). The treatment response to ICI therapy is summarized in **Table 2**. It was noted that six (15.8%) patients had discordant responses between intra- and extracranial lesions among 38 patients in whom lesions were measurable. Among these patients, six patients had brain progression while they had an extracranial response or SD. The intra- and extracranial changes of patients with measurable lesions are shown in **Figure 2**.

**Table 2:** Treatment response to ICI therapies.

Patients	Response—N (%)				ORR (%)	DCR (%)
	CR	PR	SD	PD		
Total patients (*n* = 53)	0 (0.0)	11 (20.8)	27 (50.9)	15 (28.3)	20.8	71.7
ICI monotherapy (n = 4)	0 (0.0)	0 (0.0)	3 (75.0)	1 (25.0)	0.0	75.0
ICI plus chemotherapy (n = 19)	0 (0.0)	7 (36.8)	9 (47.4)	3 (15.8)	36.8	84.2
ICI plus anti-angiogenesis (n = 12)	0 (0.0)	0 (0.0)	4 (33.3)	8 (66.7)	0.0	33.3
ICI plus chemotherapy plus anti-angiogenesis (n = 18)	0 (0.0)	4 (22.2)	11 (61.1)	3 (16.7)	22.2	83.3
Patients with measurable CNS lesions (*n* = 38)	1 (2.6)	7 (18.4)	18 (47.4)	12 (31.6)	21.0	68.4
ICI monotherapy (n = 3)	0 (0.0)	0 (0.0)	1 (33.3)	2 (66.7)	0.0	33.3
ICI plus chemotherapy (n =16)	1 (6.3)	5 (31.3)	7 (43.7)	3 (18.7)	37.5	81.3
ICI plus anti-angiogenesis (n = 7)	0 (0.0)	0 (0.0)	2 (28.6)	5 (71.4)	0.0	28.6
ICI plus chemotherapy plus anti-angiogenesis (n = 12)	0 (0.0)	2 (16.7)	8 (66.6)	2 (16.7)	16.7	83.3
Patients with measurable CNS lesions who received concurrent iRT (*n* = 9)	1 (11.1)	3 (33.3)	4 (44.5)	1 (11.1)	44.4	88.9

ICI, immune checkpoint inhibitor; CNS, central nervous system; iRT, intracranial radiotherapy; CR, complete response; PR, partial response; SD, stable disease; PD, progression disease; ORR, objective response rate; DCR, disease control rate.

**Figure 3:**
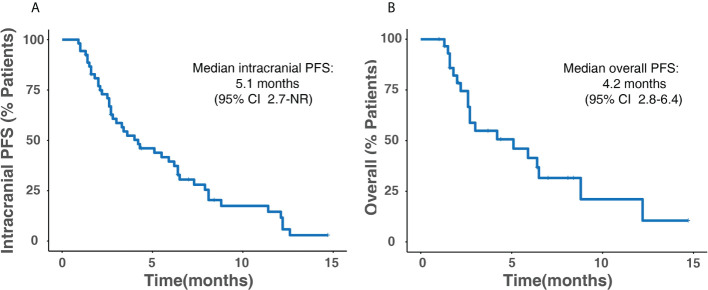
Kaplan–Meier survival analysis for all patients. **(A)** Intracranial PFS. **(B)** Overall PFS. PFS, progression-free survival; CI, confidence interval; NR, not reached.

**Figure 4:**
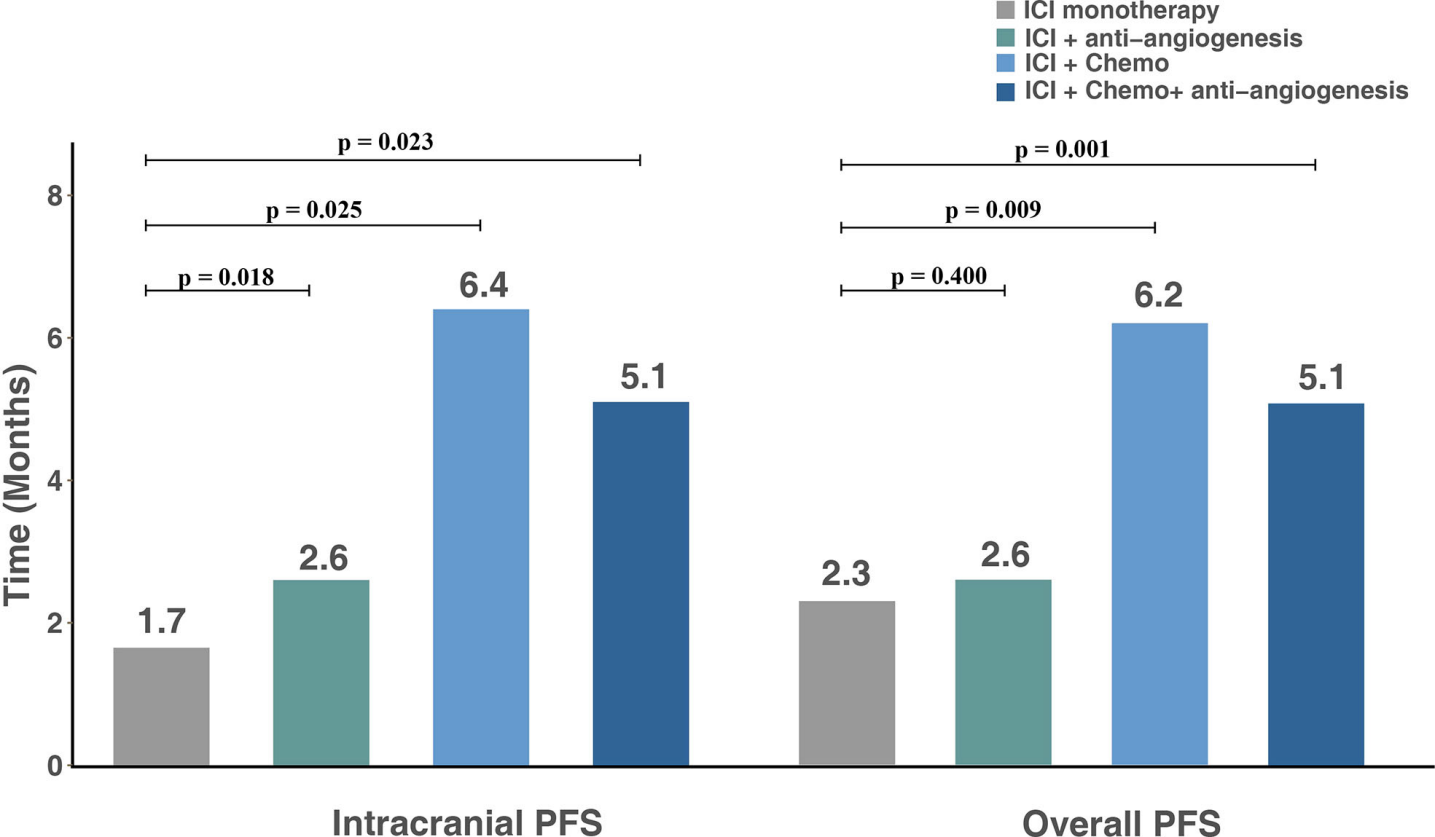
Intracranial and overall PFS for subgroups with different treatment modalities. PFS, progression-free survival; ICI, immune checkpoint inhibitor; Chemo, chemotherapy.

**Figure 5:**
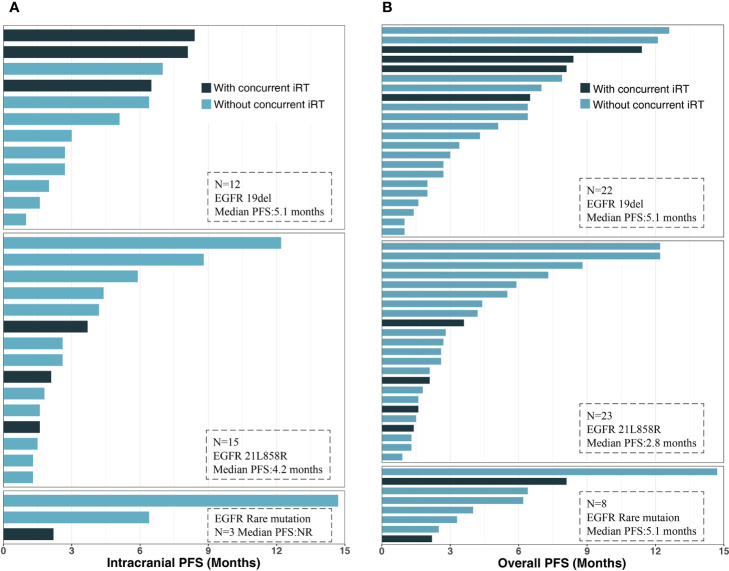
Intracranial and overall PFS for subgroups with different gene mutation types: **(A)** intracranial PFS. **(B)** Overall PFS. PFS, progression-free survival; iRT, intracranial radiotherapy; EGFR, epidermal growth factor receptor.

The median overall PFS for all patients was 4.2 months (95% CI, 2.8–6.4); after the exclusion of patients who had extracranial progression before CNS progression, 30 patients were evaluated with a median intracranial PFS of 5.1 months (95% CI, 2.7-NR) (**Figure 3**). Patients treated with ICI plus chemotherapy exhibited the longest median overall (6.2 months) and intracranial PFS (6.4 months) compared to other three treatment strategies (**Figure 4**). A previous study reported that median PFS with ICI therapy was different across patients with distinct EGFR mutation types (24). In our study, it seems that patients harboring the EGFR 21L858R mutation had shorter overall and intracranial PFS than those with the EGFR 19del mutation, but this difference did not reach statistical significance (overall PFS: 2.8 *vs*. 5.1 months, p = 0.360; intracranial PFS: 4.2 *vs*. 5.1 months, p = 0.650) (**Figure 5**).

**Figure 6:**
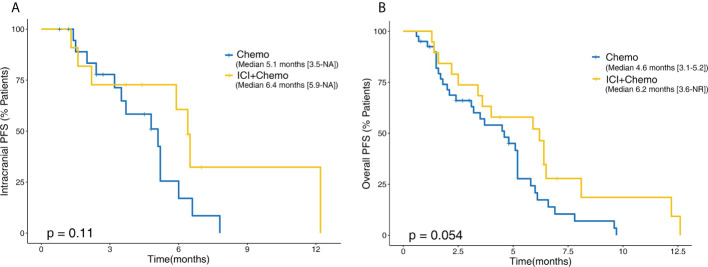
Kaplan–Meier comparative survival analysis between patients treated with chemotherapy alone and ICI plus chemotherapy. **(A)** Intracranial PFS. **(B)** Overall PFS. PFS, progression-free survival; NR, not reached.

At present, the standard of care for EGFR mutant NSCLC after EGFR-TKI failure is platinum-doublet chemotherapy. Thus, we wanted to make a comparison between salvage chemotherapy and ICI plus chemotherapy to verify whether this combination could be a promising candidate. Forty additional patients with BMs and EGFR mutations who received chemotherapy after EGFR-TKI failure were included in this analysis; the clinical characteristic of those and 19 treated with ICI plus chemotherapy is shown in **Table 3**. In the ICI plus chemotherapy cohort, there were significantly higher proportions of patients with EGFR rare mutations (p = 0.021) and more than or equal to two lines of prior systemic therapies (p = 0.001). Compared to patients treated with salvage chemotherapy, patients receiving ICI combined with chemotherapy had both longer intracranial PFS (6.4 *vs*. 5.1 months, p = 0.110) and overall PFS (6.2 *vs*. 4.6 months, p = 0.054), and these differences approached statistical significance (**Figure 6**).

**Table 3:** Comparison of clinical characteristics between patients treated with chemotherapy alone and ICI plus chemotherapy.

Characteristic	Chemo N (%)	ICI + Chemo N (%)	p value
**Age at diagnosis** <60 ≥60	28 (70.0) 12 (30.0)	11 (57.9) 8 (42.1)	0.533
**Sex** Male Female	13 (32.5) 27 (67.5)	9 (47.4) 10 (52.6)	0.415
**ECOG-PS** 0-1 2	39 (97.5) 1 (2.5)	16 (84.2) 3 (15.8)	0.179
**Smoking history** Never Smoked	33 (82.5) 7 (17.5)	17 (89.5) 2 (10.5)	0.758
**Histology** Adenocarcinoma	40 (100.0)	19 (100.0)	NA
**Molecular genotype** EGFR 19del EGFR 21L858R EGFR rare mutation	18 (45.0) 20 (50.0) 2 (5.0)	6 (31.6) 7 (36.8) 6 (31.6)	0.021
**Acquired T790M mutation** No or unknown Yes	30 (75.0) 10 (25.0)	17 (89.5) 2 (10.5)	0.345
**PD-L1 expression** Negative or unknown 1%-49% ≥50%	36 (90.0) 4 (10.0) 0 (0.0)	13 (68.4) 2 (10.5) 4 (21.1)	0.010
**High disease burden** No Yes	26 (65.0) 14 (35.0)	12 (63.2) 7 (36.8)	1.000
**Number of BMs** Single Multiple	10 (25.0) 30 (75.0)	9 (47.4) 10 (52.6)	0.156
**Symptomatic BMs** No Yes	35 (87.5) 5 (12.5)	15 (78.9) 4 (21.1)	0.641
**Prior lines of systemic therapy** 1 ≥2	26 (65.0) 14 (35.0)	3 (15.8) 16 (84.2)	0.001
**Prior intracranial RT** No Yes	17 (42.5) 23 (57.5)	10 (52.6) 9 (47.4)	0.653
**Prior EGFR-TKI response time** <10 months ≥10 months	28 (70.0) 12 (30.0)	12 (63.2) 7 (36.8)	0.820
**Prior third-generation EGFR-TKI therapy** No Yes	18 (45.0) 22 (55.0)	7 (36.8) 12 (63.2)	0.756
**Concurrent intracranial RT** No Yes	37 (92.5) 3 (7.5)	14 (73.7) 5 (26.3)	0.117
**Concurrent extracranial RT** No Yes	34 (85.0) 6 (15.0)	15 (78.9) 4 (21.1)	0.835

EGFR, epidermal growth factor receptor; ECOG-PS, Eastern Cooperative Oncology Group performance status; PD-L1, programmed death-1 ligand; BMs, brain metastases; RT, radiotherapy; TKI, tyrosine kinase inhibitor; ICI, immune checkpoint inhibitor; Chemo, chemotherapy; NA, Not applicable.

### Prognostic factors for progression-free survival

Furthermore, we evaluated the effect of different variables on PFS using univariate and multivariate Cox model analyses. All variables displaying significant correlations and trends (p < 0.150) in univariate analysis were included in multivariate analysis. For overall PFS, high disease burden (p = 0.019), receiving prior third-generation TKI treatment (p = 0.032), and a poor LIPI (p = 0.012) were independent negative predicators (**Table 4**). In addition, multiple BMs demonstrated a significant correlation with intracranial PFS (p = 0.049) (**Table 5**).

**Table 4:** Univariate and multivariate survival analyses of overall PFS.

Variables	Univariate survival analyses of overall PFS			Multivariate survival analyses of overall PFS		
	HR	95% CI	p value	HR	95% CI	*p* value
**Age at diagnosis** <60 ≥60	1.02	0.98-1.06	0.276			
**Sex** Female Male	1.09	0.6-1.99	0.775			
**ECOG-PS** 0-1 2	0.79	0.28- 2.24	0.655			
**Smoking history** Never Smoked	2.43	1.00-5.94	0.051	1.83	0.64-5.24	0.261
**Histology** Adenocarcinoma Non-adenocarcinoma	0.92	0.28-3.02	0.897			
**Molecular genotype** EGFR 19del EGFR 21L858R EGFR rare mutation	1.37 0.74	0.72-2.61 0.29-1.87	0.341 0.528			
**Acquired T790M mutation** No or unknown Yes	0.702	0.35-1.41	0.321			
**PD-L1 expression** Negative or unknown Positive	1.04	0.48- 2.26	0.928			
**High disease burden** No Yes	1.87	1-3.49	0.049	2.24	1.14-4.40	**0.019**
**Number of BMs** Single Multiple	1.54	0.84- 2.82	0.161			
**Symptomatic BMs** No Yes	1.17	0.54-2.5	0.691			
**Prior intracranial RT** No Yes	0.70	0.38-1.29	0.246			
**Prior lines of systemic therapy** 1 ≥2	1.41	0.59-3.38	0.438			
**Prior EGFR-TKI response time** <10 months ≥10 months	0.61	0.3-1.25	0.176			
**Prior third-generation EGFR-TKI therapy** No Yes	1.86	1.00- 3.45	0.049	2.16	1.07-4.37	** 0.032**
**Corticosteroid use at start of ICI treatment** No Yes	1.30	0.71-2.40	0.395			
**Combination modalities** ICI monotherapy ICI plus chemo ICI plus anti-angiogenesis ICI plus chemotherapy plus anti-angiogenesis	0.28 0.42 0.27	0.09-0.90 0.12-1.39 0.08-0.88	0.032 0.154 0.030	0.15 0.26 0.13	0.04-0.57 0.07-1.04 0.04-0.50	**0.005** 0.057 **0.003**
**Combination intracranial RT** No Yes	0.72	0.32-1.63	0.429			
**Combination extracranial RT** No Yes	1.91	0.92-3.96	0.082	2.06	0.87-4.88	0.100
**dNLR** <3 ≥3	1.34	0.64-2.81	0.446			
**LDH** <ULN ≥ULN	1.46	0.79-2.68	0.223			
**LIPI** Good (0) and intermediate (1) Poor (2)	3.39	1.50-8.23	0.007	3.86	1.35-11.07	**0.012**

Bold values indicates significant results with p < 0.05. HR, hazard ratio; CI, confidence interval; BMs, brain metastases. EGFR, epidermal growth factor receptor; ECOG-PS, Eastern Cooperative Oncology Group performance status; PD-L1, programmed death-1 ligand; RT, radiotherapy; TKI, tyrosine kinase inhibitor; ICI, immune checkpoint inhibitor; dNLR, derived neutrophil-to-lymphocyte ratio; LDH, lactate dehydrogenase; ULN, upper limit of normal; LIPI, immune prognostic index.

**Table 5:** Univariate and multivariate survival analyses of intracranial PFS.

Variables	Univariate survival analyses of intracranial PFS			Multivariate survival analyses of intracranial PFS		
	HR	95% CI	*p* value	HR	95% CI	*p* value
**Age at diagnosis** <60 ≥60	1.01	0.96-1.07	0.683			
**Sex** Female Male	1.01	0.39-2.6	0.988			
**KPS** 0-1 2	0.60	0.14-2.65	0.503			
**Smoking history** Never Smoked	1.89	0.54-6.63	0.32			
**Histology** Adenocarcinoma Non- adenocarcinoma	1.88	0.42-8.51	0.413			
**Molecular genotype** EGFR 19del EGFR 21L858R EGFR rare mutation	1.29 0.30	0.49-3.43 0.04-2.53	0.610 0.269			
**Acquired T790M mutation** No or unknown Yes	0.81	0.28-2.31	0.693			
**PD-L1 expression** Negative or unknown Positive	2.26	0.72- 7.07	0.163			
**High disease burden** No Yes	2.58	0.98-6.74	0.054	2.13	0.58-7.91	0.257
**Number of BMs** Single Multiple	2.41	0.88- 6.63	0.088	3.90	1.00-15.12	**0.049**
**Symptomatic BMs** No Yes	0.97	0.27-3.51	0.973			
**Prior intracranial RT** No Yes	0.62	0.23-1.65	0.339			
**Prior lines of systemic therapy** 1 ≥2	1.34	0.38-4.75	0.646			
**Prior EGFR-TKI response time** <10 months ≥10 months	0.47	0.17-1.28	0.138	0.279	0.08-1.03	0.056
**Prior third-generation EGFR-TKI therapy** No Yes	2.46	0.85-7.12	0.096	2.32	0.63-8.63	0.208
**Corticosteroid use at start of ICI treatment** No Yes	0.97	0.36-2.57	0.946			
**Combination modalities** ICI monotherapy ICI plus chemotherapy ICI plus anti-angiogenesis ICI plus chemotherapy plus anti-angiogenesis	0.06 0.16 0.04	0.09-0.90 0.12-1.39 0.08-0.88	0.005 0.055 0.003	0.02 0.09 0.02	0.00-0.18 0.01-0.86 0.00-0.16	**< 0.001** **0.036** **< 0.001**
**Combination intracranial RT** No Yes	0.36	0.08-1.57	0.173			
**Combination extracranial RT** No Yes	1.28	0.42-3.94	0.666			
**dNLR** <3 ≥3	2.25	0.69-7.37	0.18			
**LDH** <ULN ≥ULN	1.73	0.7-4.31	0.236			
**LIPI** Good (0) and intermediate (1) Poor (2)	2.45	0.75-8.00	0.138	2.063	0.47-9.00	0.336

Bold values indicates significant results with p < 0.05. HR, hazard ratio; CI, confidence interval; BMs, brain metastases; EGFR, epidermal growth factor receptor; ECOG-PS, Eastern Cooperative Oncology Group performance status; PD-L1, programmed death-1 ligand; RT, radiotherapy; TKI, tyrosine kinase inhibitor; ICI, immune checkpoint inhibitor; dNLR, derived neutrophil-to-lymphocyte ratio; LDH, lactate dehydrogenase; ULN, upper limit of normal; LIPI, immune prognostic index.

**Figure 7:**
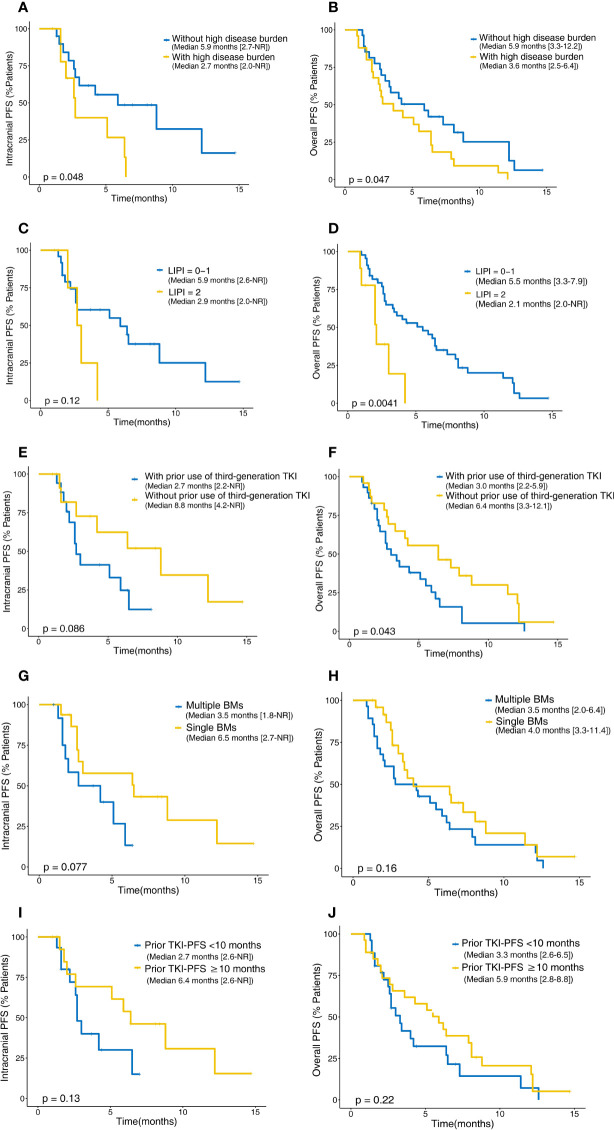
Kaplan–Meier analysis for intracranial and overall PFS in patients with different risk factors. Intracranial **(A)** and overall PFS **(B)** between patients with and without high disease burden. Intracranial **(C)** and overall PFS **(D)** between patients with and without poor LIPI. Intracranial **(E)** and overall PFS **(F)** between patients with and without prior usage of third-generation EGFR-TKI. Intracranial **(G)** and overall PFS **(H)** between patients with and without multiple BMs. Intracranial **(I)** and overall PFS **(J)** between patients with prior TKI-PFS <10 months and ≥10 months. PFS, progression-free survival; NR, not reached; LIPI, immune prognostic index; EGFR, epidermal growth factor receptor; TKI, tyrosine kinase inhibitor; BMs, brain metastases.

Survival analysis was then performed according to the factors described above. Patients with high disease burden had significantly shorter overall PFS (3.6 *vs*. 5.9 months, p = 0.047) and intracranial PFS (5.9 *vs*. 2.7 months, p = 0.048) than those without disease burden. A shorter median overall PFS of 2.1 months was observed for patients who had poor LIPI, while the median overall PFS was 5.5 months for those having a good or intermediate LIPI (p = 0.004). In addition, patients who had been treated with prior third-generation EGFR-TKI therapy were significantly associated with a poor overall PFS (3.0 months *vs*. 6.4 months, p = 0.043). Patients who had a poor LIPI (2.9 *vs*. 5.9 months, p = 0.120), prior third-generation EGFR-TKI therapy (8.8 *vs*. 2.7 months, p = 0.086), or multiple BMs (3.5 *vs*. 6.5 months, p = 0.077) had shorter intracranial PFS; although these differences were not statistically significant, there was a trend toward significance. It has been previously reported that the PFS of patients with prior EGFR-TKI therapy can serve as a predictor of efficacy for ICI therapy at a 10-month cutoff value (25). In our study, longer overall PFS (5.9 *vs*. 3.3 months, p = 0.22) and intracranial PFS (6.4 *vs*. 2.7 months, p = 0.13) were observed in patients with PFS of prior EGFR-TKI therapy greater than or equal to 10 months, although these differences did not reach statistical significance (**Figures 7A–H**).

## Discussion

EGFR-TKI therapy is the standard first-line treatment in patients with EGFR mutations with BMs; however, the treatment options of those patients whose disease progresses after EGFR TKI therapy are limited. In this study, we found that among all ICI-containing therapies, ICI combined with chemotherapy had better CNS efficacy and it led to longer intracranial PFS and overall PFS compared to salvage chemotherapy, which suggests that this combined treatment strategy is effective and may be considered as a treatment option for these refractory patients.

The occurrence of BMs, especially untreated or active BMs, often prevents participation in clinical trials for novel systemic therapy owing to concerns about the potential drug exclusion by the BBB. However, the presence of lymph vessels linking the brain and deep cervical lymph nodes allows immune cells to pass between the brain and peripheral system (26, 27). In addition, a preclinical study demonstrated that ICI could induce the priming and trafficking of CD8+ T cells from extracranial tumors to the brain, contributing to potent intracranial treatment efficacy for BMs in melanoma (28). A phase 2 prospective study suggested that a PD-1 inhibitor had CNS efficacy, with an intracranial ORR of 29.7% and PFS of 2.3 months in PD-L1-positive NSCLC patients with untreated BMs (16). Moreover, a series of retrospective studies have reported superior CNS efficacy of ICI therapy compared to ICI-naïve therapy (29–31). Nevertheless, the vast majority of patients in these studies have wild-type EGFR, and data regarding the intracranial efficacy of ICI therapy in patients with EGFR mutations with BMs are rare. Our study focused on this subpopulation that progressed with prior EGFR-TKI therapy because EGFR-TKI is still the first-line treatment option for those with EGFR mutations. We found that the intracranial ORR of ICI therapy was 21.0% and that the intracranial PFS was 5.1 months in all patients with measurable BMs. For patients receiving ICI plus chemotherapy, the efficacy data are encouraging, with an intracranial ORR of 37.5% and a median intracranial PFS of 6.4 months. These results were superior to those of a previous study reported by Goldberg et al. (16); the likely reason for this is the combination strategy of ICI and chemotherapy evaluated in our study. In addition, a small proportion of patients received concurrent brain radiotherapy, which further improved disease control. However, ICI plus chemotherapy plus antiangiogenic agents had an intracranial ORR of 16.7%, which was lower than that of the combination of ICI and chemotherapy. This may be because the proportion of positive PD-L1 patients and the proportion of low LIPI patients were both higher in the ICI plus chemotherapy group, contributing to its superior efficacy (**Supplement Table**).

We also found that there was no intracranial response in patients receiving ICI monotherapy or ICI plus antiangiogenic agents. This may not be surprising for ICI monotherapy because patients with EGFR mutations had an extremely poor response to ICI monotherapy, and a previous study reported an ORR of only 3.6% (32). Additionally, most patients had ≥2 prior therapies (86.8%) in our study, which means that tumors may have strong drug resistance. Therefore, the addition of antiangiogenic agents alone may increase the efficacy of ICI to a limited extent and may even not produce any synergy. The important role of chemotherapy in ICI-containing strategies against heavily pretreated NSCLC was revealed in comparisons of ICI plus chemotherapy and strategies without chemotherapy. Moreover, the discordance of treatment response between intracranial and extracranial lesions should also be noted. The discordance rate was 15.8% in our study, which was consistent with other previous studies ranging from 12.7% to 36.4% (16, 33, 34). The reason for the discordant outcome may be the difference in the tumor microenvironment between primary tumors and BMs. Previous studies have shown that there was significant disagreement of both PD-L1 expression and CD8+ T-cell infiltration that are critical contributors to the efficacy of ICI between BMs and matched primary tumors in NSCLC (35, 36). Therefore, the identification of the heterogeneity between primary tumors and metastases may help us better tailor the treatment of patients with metastases.

Currently, platinum-containing chemotherapy is the standard salvage therapy for EGFR-mutant NSCLC patients who failed EGFR-TKI therapy. Our study first compared both intracranial and overall PFS between ICI plus chemotherapy and chemotherapy alone in the BM subpopulation. Patients receiving ICI combined with chemotherapy had both longer intracranial PFS (6.4 *vs*. 5.1 months, p = 0.110) and overall PFS (6.2 *vs*. 4.6 months, p = 0.054) than those receiving chemotherapy, although the differences did not reach statistical significance, which could be due to the small sample size. A previous phase 2 study evaluated ICI plus chemotherapy as second-line treatment in advanced patients with EGFR mutations, and the median overall PFS in this study was slightly longer than ours (7.0 *vs*. 6.2 months) (21). The fewer prior lines of therapy in this study might explain the survival difference. Another phase 3 study, ORIENT-31, evaluated three ICI regimens, including ICI plus chemotherapy plus antiangiogenic agents, ICI plus chemotherapy, and chemotherapy, and the median overall PFS times were 6.9, 5.6, and 4.3 months, respectively. The data of ICI plus chemotherapy and chemotherapy in our study are generally consistent with these results; however, the overall PFS of the triple combination in our study was lower than that of the other combinations (5.1 *vs*. 6.9 months) (37). The low proportion of PD-L1-positive patients and the small sample size of our study may explain this difference. Consequently, the combination of ICI and chemotherapy may represent a new option for patients with EGFR mutations with BMs for whom EGFR-TKIs have failed; however, additional studies are needed to fully demonstrate the benefits.

The identification of prognostic factors is useful to predict clinical outcomes based on tumor and patient characteristics. For patients with EGFR mutations, the type of EGFR mutation may influence the outcomes of patients treated with ICI. The IMMUNOTARGET study suggested that the EGFR 21L858R mutation was associated with favorable outcomes compared to the EGFR 19del mutation (24). In our study, patients with the EGFR 19del mutation had longer PFS than patients with the EGFR 21L858R mutation, which seems inconsistent with a previous study. However, the vast majority of patients in our studies were treated with combination therapies, while patients in the IMMUNOTARGET study received only ICI monotherapy; moreover, there was considerable heterogeneity in trial designs and populations between these two studies, which precludes a direct comparison of these results. Another tumor characteristic affecting patient outcomes is disease burden. We found that a high disease burden, defined as three or more metastatic organs, was an independent negative predictor for both intracranial and extracranial PFS, which is consistent with previous studies (33).

The LIPI is obtained from serum LDH and peripheral lymphocytes and neutrophils, reflecting the patient’s immune system status. In our study, we observed that a poor LIPI was significantly associated with shorter overall PFS. This is consistent with the literature (23, 38, 39). Thus, the LIPI could be a useful predictor of the outcomes of ICI therapy. In addition, the baseline prior EGFR-TKI therapy may also influence the efficacy of current salvage therapy. Patients with previous third-generation EGFR-TKI therapy had significantly shorter PFS than those without this therapy in our study. Although the third-generation EGFR-TKI osimertinib has demonstrated potent CNS efficacy with an intracranial PFS of 39.7 in T790M-positive NSCLC patients with BMs (40), once tumors have progressed and acquired resistance to the drug, the prognosis will be poor. Moreover, we observed that patients with prior EGFR-TKI with a PFS of less than 10 months tended to have shorter PFS with ICI therapy, which is consistent with the study reported by Bai et al. (41).

Our study has several limitations. First, this was a retrospective analysis with a small number of patients. Second, patients did not receive treatment with the same EGFR-TKI and ICI types and a substantial proportion of patients had unknown PD-L1 expression and T790M mutation due to incomplete medical records, which may affect the outcomes with ICI therapy. Third, we lacked molecular analysis for BMs because there may be inconsistencies in both EGFR mutation status and PD-L1 expression between primary and BMs, but the collection of brain tissue samples is difficult in real-world practice. Last, the OS data were not mature at the time of the last follow-up. Given these limitations, a large-scale prospective study is required to validate our results.

In summary, the findings of this retrospective study demonstrated the promising intracranial efficacy of ICI plus chemotherapy in patients with EGFR-mutant NSCLC with BMs who experienced prior EGFR-TKI failure. Both intracranial and overall PFS were longer with this combination than with chemotherapy, which suggests that it may become a treatment option for these patients.

## Data availability statement

The original contributions presented in the study are included in the article/**Supplementary Material**. Further inquiries can be directed to the corresponding author.

## Ethics statement

This study was reviewed and approved by The Ethics Review Board of Shandong Cancer Hospital. The ethics committee waived the requirement of written informed consent for participation.

## Author contributions

XM conceived of the review and edited the manuscript. FR collected the data. SZ analyzed the data and drafted the article. All authors contributed to the article and approved the submitted version.

## Funding

The study was funded by National Natural Science Foundation of China (81972796 and 82272845), Natural Science Foundation of Shandong Province (ZR2019MH010 and ZR2019MH289), Beijing Bethune Charitable Foundation (flzh202107), Wu Jieping Medical Foundation (320.6750.2020-12-10), CSCO-Haosen Foundation (Y-HS202102-0089), and CSCO-Xinda Foundation (Y-XD202001-0008).

## Conflict of interest

The authors declare that the research was conducted in the absence of any commercial or financial relationships that could be construed as a potential conflict of interest.

## Publisher’s note

All claims expressed in this article are solely those of the authors and do not necessarily represent those of their affiliated organizations, or those of the publisher, the editors and the reviewers. Any product that may be evaluated in this article, or claim that may be made by its manufacturer, is not guaranteed or endorsed by the publisher.
