# Supplementary material for: Efficacy of immune checkpoint inhibitor therapy in EGFR mutation-positive patients with NSCLC and brain metastases who have failed EGFR-TKI therapy
Source: Front Immunol. 2022 Sep 27;13:955944. doi: 10.3389/fimmu.2022.955944 (PMC9552846; doi:10.3389/fimmu.2022.955944)
Supplement: Supplementary file 1 [file Table_1.docx]

**Supplement Table**

The comparison of characteristic between patients treated with ICI plus chemotherapy and ICI plus chemotherapy plus anti-angiogenesis

| **Characteristic** | **ICI + Chemo**  **N (%)** | **ICI + Chemo + Anti-angiogenesis**  **N (%)** | **p** |
| --- | --- | --- | --- |
| **Age at diagnosis**  < 60  ≥ 60 | 11 (57.9)  8 (42.1) | 14 (77.8)  4 (22.2) | 0.347 |
| **Sex**  Male  Female | 10 (52.6)  9 (47.4) | 10 (55.6)  8 (44.4) | 0.415 |
| **ECOG-PS**  0-1  2 | 16 (84.2)  3 (15.8) | 18 (100.0）  0 (0.0) | 0.088 |
| **Histology**  Adenocarcinoma  Other | 19 (100.0)  0 (0.0) | 15 (83.3)  2 (11.1) | 0.179 |
| **PD-L1 expression**  Negative or Unknown  1-49%  ≥ 50% | 13 (68.4)  2 (10.5)  4 (21.1) | 17 (94.4)  0 (0.0)  1 (5.6) | **0.116** |
| **LIPI**  0-1  2 | 19 (100.0)  0 (0.0) | 12 (66.7)  6 (33.3) | **0.021** |
| **High disease burden**  No  Yes | 12 (63.2)  7 (36.8) | 9 (50.0)  9 (50.0) | 1.000 |
| **Number of BMs**  Single  Multiple | 10 (25.0)  30 (75.0) | 9 (47.4)  10 (52.6) | 0.634 |
| **Prior lines of systemic therapy**  median  IQR | 2  2-4 | 2  2-3 | 0.658 |
| **Prior intracranial RT**  No  Yes | 10 (52.6)  9 (47.4) | 12 (66.7)  6 (33.3) | 0.593 |
| **Prior TKI response time**  < 10 months  ≥ 10 months | 12 (63.2)  7 (36.8) | 8 (44.4)  10 (55.6) | 0.417 |
| **Prior third-generation EGFR-TKI therapy**  No  Yes | 12 (63.2)  7 (36.8) | 9 (50.0)  9 (50.0) | 0.634 |
| **Concurrent intracranial RT**  No  Yes | 14 (73.7)  5 (26.3) | 14 (77.8)  4 (22.2) | 0.117 |

BMs: Brain metastases. EGFR: Epidermal growth factor receptor; ECOG-PS: Eastern Cooperative Oncology Group performance status; PD-L1: Programmed death-1 ligand; RT: Radiotherapy; TKI: Tyrosine kinase inhibitor; ICI: Immune checkpoint inhibitor; LIPI: Immune prognostic index
